# Single Molecule Study of Hydrogen Bond Interactions Between Single Oligonucleotide and Aerolysin Sensing Interface

**DOI:** 10.3389/fchem.2019.00528

**Published:** 2019-07-31

**Authors:** Meng-Yin Li, Ya-Qian Wang, Yao Lu, Yi-Lun Ying, Yi-Tao Long

**Affiliations:** ^1^School of Chemistry and Molecular Engineering, East China University of Science and Technology, Shanghai, China; ^2^State Key Laboratory of Analytical Chemistry for Life Science, School of Chemistry and Chemical Engineering, Nanjing University, Nanjing, China

**Keywords:** single-molecule interface, oligonucleotide, nanopore, hydrogen bond, nanoconfinement

## Abstract

The aerolysin nanopore displays a charming sensing capability for single oligonucleotide discrimination. When reading from the electrochemical signal, stronger interaction between the aerolysin nanopore and oligonucleotide represent prolonged duration time, thereby amplifying the hidden but intrinsic signal thus improving the sensitivity. In order to further understand and optimize the performance of the aerolysin nanopore, we focus on the investigation of the hydrogen bond interaction between nanopore, and analytes. Taking advantage of site-direct mutagenesis, single residue is replaced. According to whole protein sequence screening, the region near K238 is one of the key sensing regions. Such a positively charged amino acid is then mutagenized into cysteine and tyrosine denoted as K238C, and K238Y. As (dA)_4_ traverses the pores, K238C dramatically produces a six times longer duration time than the WT aerolysin nanopore at the voltage of +120 mV. However, K238Y shortens the dwell time which suggests the acceleration of the translocation causing poor sensitivity. Referring to our previous findings in K238G, and K238F, our results suggest that the hydrogen bond does not dominate the dynamic translocation process, but enhances the interaction between pores and analytes confined in such nanopore space. These insights give detailed information for the rational design of the sensing mechanism of the aerolysin nanopore, thereby providing further understanding for the weak interactions between biomolecules and the confined space for nanopore sensing.

## Introduction

The biological nanopore is a promising sensing tool for single molecule detection under an external ionic flux (Kasianowicz et al., 1996; Wang et al., 2013a; Cao and Long, 2018). Due to its high sensing ability for the molecules in nanometer scale, the biological nanopore has been developing especially in life science over the past three decades (Stefureac et al., 2006; Gu and Shim, 2010; Pastoriza-Gallego et al., 2011; Wen et al., 2011; Wang et al., 2015). Such a single-molecule sensing interface originating from a single protein provides a well-confined nano-space for restricting a single entity reading from electrochemical signal (Bayley and Cremer, 2001; Deamer and Branton, 2002; Ying et al., 2018). In order to optimize the current and temporal resolution for different individuals, various membrane proteins, and toxins have been studied as nanopores, such as α-hemolysin (Kasianowicz et al., 1996; Ying et al., 2013), MspA (Butler et al., 2008), aerolysin (Cao et al., 2016a), phi29 DNA-packaging nanomotor (Wendell et al., 2009), SP1 (Wang et al., 2013b), OmpG (Fahie et al., 2015), NfpAB (Singh et al., 2012), ClyA (Soskine et al., 2012), FhuA (Mohammad et al., 2012), and CsgG (Brown and Clarke, 2016). Volume restriction and dynamic interaction between analyte and nanopore are considered as the two dominant factors determining the sensitivity and selectivity of the biological nanopore sensing (Ying et al., 2018). The confinement of nanopore accommodates the single analyte for the further characteristic interactions inside the nanopore, leading to the distinguishable ionic signature. For example, the dsDNA and proteins require large nanopore (d > 2 nm) such as ClyA (Soskine et al., 2012), phi29 (Wendell et al., 2009), and FhuA (Mohammad et al., 2012) for efficient non-covalent interactions, while the ssDNA, peptide and small polymers ask small nanopores (<2 nm) such as MspA (Butler et al., 2008), CsGg (Brown and Clarke, 2016), and aerolysin (Cao et al., 2016b) for better pore-analyte interactions. In recent years, due to the special geometric structure with long β-barrel and many charged amino acid residues inside the pore lumen, aerolysin nanopore exhibited charming capability for molecule sensing, such as the discrimination of DNA or peptide with different lengths (Cao et al., 2016a; Wang et al., 2017; Piguet et al., 2018), direct readout of single molecule size of PEG and single nucleobase variations in an oligonucleotides (Baaken et al., 2015; Cao et al., 2017), even real-time monitoring of methylcytosine under serum condition (Yu et al., 2017).

As the single analyte driven inside the nanopore, the sensing region of aerolysin is responsible for most of the interaction for the whole duration. For example, our recent studies have shown that K238 site of aerolysin nanopore has sensitive interactions with oligonucleotide (Cao et al., 2018). Moreover, the replacement of positively charged lysine in 238 site with negatively charged Glutamic acid (K238E) prolongs the translocation time about 20 times for negatively charged (dA)_4_ comparing to that of wild-type (WT) aerolysin (Wang et al., 2018a). The electrostatic repulsion between K238E and oligonucleotides induces the back and forth motion of (dA)_4_, resulting in current variations during the single blockage event. Other mutations such as K238F and K238G also demonstrate that K238 site greatly contributed to the temporal resolution for oligonucleotides detection due to the sensitive non-covalent interactions (Wang et al., 2018b). However, the questions still remain for the dominated type of non-covalent interaction inside aerolysin nanopore as sensing the oligonucleotide.

**Figure 1:**
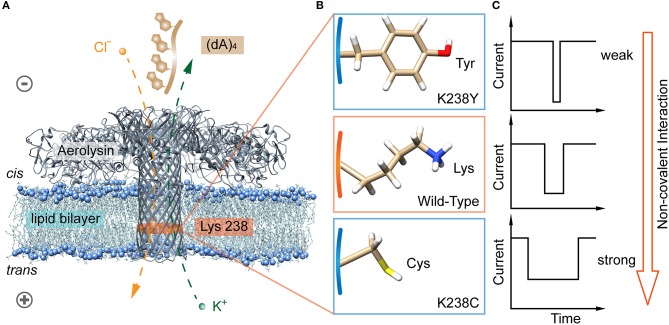
Identification of hydrogen bond interaction at K238 site of the aerolysin nanopore for single oligonucleotides sensing. **(A)** All-atom model of aerolysin heptamer inserted into lipid bilayer to form the nanopore sensing system (PDB: 5JZT). The orange band represents the sensing region near 238 site. **(B)** Amino acids structure on 238 site of K238Y, WT, and K238C aerolysin, respectively. **(C)** The modeled current traces of (dA)_4_ with K238Y, WT, and K238C aerolysin, respectively. Due to the pH of solution is 8.0, the lysine is protonated in **(B)**. The images of aerolysin system and amino acid structure are created by UCSF Chimera (Pettersen et al., 2004).

Among the non-covalent interactions, hydrogen bond is typical and considered in the following content. We further designed two mutant aerolysin nanopores (K238C and K238Y) to study the role of hydrogen-bond interaction at 238 site for the translocation behaviors of oligonucleotide (Figures 1A,B). Compared to K238F and K238G in our previous study (Wang et al., 2018b), K238Y and K238C possess hydroxyl, and sulfhydryl group on the amino acid residues, respectively. In principle, these two mutations could site-directly increase the possibility for forming hydrogen bonds during the dynamic interaction process with oligonucleotides (Luscombe et al., 2001). The results show that K238C mutant nanopore produces extremely long blockage duration especially at the applied voltage of +120 mV, which is approximately six times longer than WT aerolysin, and about ~1.3 ms longer than K238G mutant. Furthermore, the duration of oligonucleotide with K238Y is about ~1 ms longer than that inside K238F (Figure 1C). Therefore, the hydrogen bond is one of the crucial factors for the oligonucleotide-pore interactions. This study could guide us to sophisticate design the sensing interface of aerolysin for further practical application on microRNA detection, DNA damage, epigenetic modifications, and even examine the activity of nucleases.

## Materials and Methods

### Chemicals and Reagents

Trypsin-agarose, trypsin-EDTA, decane (anhydrous, ≥99%), Na_3_PO_4_, HCl, NaCl, and imidazole are purchased from Sigma-Aldrich Co., Ltd. (St. Louis, MO, USA). 1, 2-diphytanoyl-snglycero-3-phosphocholine (chloroform, ≥99%) is purchased from Avanti Polar Lipids, Inc. (Alabaster, Al, USA). All polynucleotides samples are synthesized and HPLC-purified by Sangon Biotech Co., Ltd. (Shanghai, China). All reagents and materials are of analytical grade. Yeast extract and peptone are purchased from OXOID Co., Ltd. (Basingstoke, UK). Glycerinum is purchased from Amresco, Inc. (Atlanta, GA, USA). IPTG is purchased from Inalco SpA, Inc. (Milano, Italy). BL21 [DE3] pLysS *E. coli* is purchased from TIANGEN Co., Ltd. (Beijing, China). The pET22b-proaerolysin plasmid are synthesized, and HPLC-purified by Genewiz, Inc. (Suzhou, China). All solutions are prepared using ultrapure water (18.2 MΩ cm at 25°C) from a Milli-Q system (Billerica, MA, USA).

### Proaerolysin Production

The preoaerolysin productions of K238C and K238Y are according to the previous studies (Iacovache et al., 2011; Wang et al., 2018a). Note that mercaptoethanol was added to the K238C aerolysin nanopore to protect the cysteine from oxidation.

### Single Molecule Measurement

The formation of mutant aerolysin nanopore is described in our previous study (Wang et al., 2018a). Both compartments of the recording chamber contain 1.0 mL of 1.0 M KCl, 10 mM Tris, pH 8.0, with 1.0 mM EDTA. The potential is applied using Ag/AgCl electrodes. About 1.0 μL monomeric aerolysin (~1.5 μg/mL) is added to the cis chamber to form the pore. The oligonucleotide is added to the *cis* compartment to a final concentration of 2.0 μM. All the nanopore experiments are conducted at 24 ± 2°C. All nanopore experiments for each mutant aerolysin were performed at least 3 separated measurements.

### Data Acquisition and Analysis

The current recordings are performed with a patch clamp amplifier (Axon 200B equipped with a Digidata 1440A A/D converter, Molecular Devices, USA). The amplified signal (arising from the ionic current passing through the pore) is sampled at 100 kHz and low-pass filtered at 5 kHz through the Clampex 10.7 software (Molecular Devices, USA). The data analysis is performed by using Mosaic software (Balijepalli et al., 2014; Forstater et al., 2016), and Origin-Lab 8.0 (Origin-Lab Corporation, Northampton, MA).

## Results and Discussions

**Figure 2:**
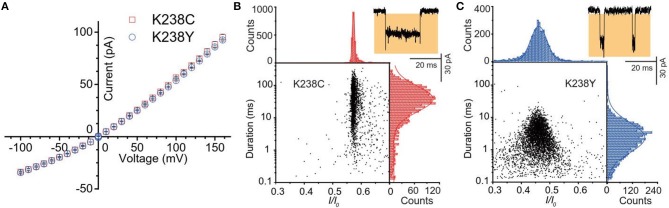
**(A)** Current-voltage curves of K238C (red) and K238Y (blue). The error-bars are from three independent mutant aerolysin nanopore experiments. Scatter plots of (dA)_4_ along with related *I/I*_0_ and duration histograms by K238C **(B)**, and K238Y **(C)** respectively at +120mV. *I/I*_0_ histograms were fitted to single Gaussian function. The log value of duration time histograms were fitted to single Gaussian function.

The aerolysin produced by proaerolysin is able to self-assemble and oligomerize as a heptameric ring-like structure that inserts into the membrane to form a nanoscale pore (Iacovache et al., 2016). As illustrated in Figure 1A, the orange-banded region that near the positively charged lysine-238 (K238) site is considered as one of the sensing regions due to its relatively small diameter and special potential distribution under an applied voltage (Wang et al., 2018b). To estimate the influences of hydrogen bonds between the amino acid residues inside the pore, and oligonucleotides on its translocation behaviors, the positively charged lysine is mutated into neutral amino acid residues of cysteine (C), and tyrosine (Y) that owns sulfhydryl and hydroxy, respectively (Figure 1B). Note that there are many serine (S) inside the aerolysin lumen, especially in its sensing regions. To decrease the effects of other amino acids nearby, the K238S is not performed. As shown in Figure 2A, two mutant pores exhibit the almost same open pore current response at biased potential ranging from 0 mV to +160 mV. The open pore currents with standard error (S.D.) of ~2 pA indicate their good structure stability under the experimental conditions. Previous studies have shown that the (dA)_4_ generates the longest translocation durations with the narrowest peak width of blockage current histogram in WT aerolysin among (dA)_2_ to (dA)_10_ (Cao et al., 2016a), which suggests the most stable and uniform behavior during every individual (dA)_4_ translocating through aerolysin. This phenomenon might be related to the optimal spatial filling of (dA)_4_ within the sensing region of aerolysin (Li et al., 2018), leading to the strong interaction between (dA)_4_, and aerolysin lumen. Therefore, we chose (dA)_4_ as the model analyte to study the interaction between nanopore, and oligonucleotide.

Firstly, we statistically analyzed the current signals produced by (dA)_4_ traversing through mutant aerolysin at +120 mV. Our previous study has demonstrated that there are two kinds of events resulting from two dynamic motions of oligonucleotides at the nanopore sensing interface, which are transient bumping events at the pore entrance, and threading events (Cao et al., 2016a). The bumping event originates from the oligonucleotides entering the aerolysin nanopore then eventually returning to the *cis* solution due to the energy barrier at the entrance, which generates the constant current amplitude with the increasing voltage (Figures S1, S2). We excluded these bumping events in our further statistical analysis since this study focuses on the interaction of an oligonucleotide traversing through the aerolysin. As shown in Figure 2B, the (dA)_4_ experiences the duration time of 3.05 ± 0.17 ms inside K238Y, while it sojourns a longer duration time of 29.69 ± 2.19 ms at +120 mV in K238C. The duration time of oligonucleotide is about six times longer than that of WT. Moreover, the oligonucleotides exhibit substantial translocation events inside K238C with Gaussian fitting of *I/I*_0_ = 0.58 ± 0.01 (*I/I*_0_, *I* represents the residual current, while *I*_0_ is the open pore ionic current). The full width at half maximum (FWHM) of Gaussian distribution is 0.015. In contrast, K238Y produces smaller *I/I*_0_ value which is concentrated at 0.50 ± 0.03 with the FWHM of 0.09 (Figure 2C). The smaller FWHM in K238C suggests a more uniform behavior of (dA)_4_ inside K238C aerolysin and a stronger interaction between them. Conferring to the previous studies of mutant aerolysin along with WT (Wang et al., 2018a), we have the order of *I/I*_0_ value as K238C > K238G> WT > K238Y ~ K238F. The *I/I*_0_ value shows an order of side chain volume, which verifies the existence of volume effect. Because the amino acid Y merely have a hydroxyl group difference compared with F, so the *I/I*_0_ value of K238Y and K238F is almost identical.

**Figure 3:**
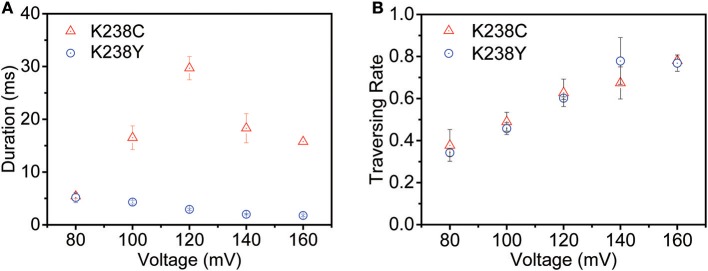
The voltage-dependent of **(A)** duration time and **(B)** threading rate plotted against the voltage for (dA)_4_ by K238C (red), and K238Y (blue) aerolysin, respectively. The applied voltage ranging from +80mV to +160mV at 20mV increments. The error-bars indicated standard deviation from data derived from three independent experiments.

The voltage-dependent experiments were then conducted to further understand the non-covalent interaction between pore and analyte. The exponentially decreased duration time with the increasing voltages suggests that (dA)_4_ translocate through the K238Y mutant pore (Figure S3). Moreover, the duration time of K238Y is lower than that of WT aerolysin at applied potential from +80 mV to +160 mV, indicating a weaker interaction of (dA)_4_ with tyrosine compared with lysine. However, the K238C exhibits exactly different experimental phenomenon (Figure 3A and Figure S4). When the applied voltage lower than +120 mV, the duration time increases with the increasing voltage. However, when the voltage is higher than +120 mV, the duration time decreases. On the basis of the previous results (Breton et al., 2013), the +120 mV can be regarded as the threshold voltage (V_T_) for the translocation of oligonucleotide. If the applied voltage is higher than V_T_, the oligonucleotide could traverse through the K238C mutant pore. More importantly, K238C and K238Y behave similar threading rate which suggest the barrier for the entrance of oligonucleotide into the aerolysin is similar as substituting the amino acid of C and Y at K238 site (Figure 3B). Here, the threading rate represents the ratio of threading events to all the events. Therefore, the evidently different duration time of (dA)_4_ in the K238C, and K238Y mutant aerolysin indeed depends on the interactions at 238 of aerolysin.

Since K238C and K238Y possess sulfhydryl and hydroxyl, respectively, they are designed to increase the possibility for forming hydrogen bonds with the oligonucleotide. As to WT aerolysin, the positively charged lysine is protonated in pH 8.0 solution, which undergoes strong electrostatic interactions with phosphorylate group of oligonucleotides. However, regarding to K238C, the cysteine is a strong hydrogen acceptor, which increases the strength of hydrogen bonds between the pore, and oligonucleotides but weaken the electrostatic interaction in 238 site. Note that the pKa for the sulfydryl of cysteine is about 8.0, suggesting that about half of cysteine should be negatively charged inside the pore at the experimental condition (pH 8.0). However, the control experiment at pH 7.0 for (dA)_4_ in K238C aerolysin shows almost same translocation during at each applied voltage (Figure S5), suggesting the same state for sulfydryl of cysteine inside the pore at pH 8.0, and 7.5 that remains neutral. This result indicates that the pKa for cysteine inside a confined environment is different from that in bulk conditions. Moreover, our previous study shows that oligonucleotide owns a long duration of ~28.4 ms in K238G at +120 mV, which is slightly shorter than that of K238C (~29.7 ms). Therefore, the hydrogen bond interaction could enhance the total non-covalent interaction between pore and oligonucleotides, but not the determined effect for the whole translocation process in K238C mutant aerolysin nanopore.

Regarding to K238Y, its side chain contains benzene ring with a hydroxyl. Our previous work has demonstrated that the K238F where phenylalanine (F) only contains benzene ring produced a much shorter duration for (dA)_4_. The hydrophobic benzene ring of K238Y and K238F probably repulse the water inside the lumen. Therefore, the amino acid at 238 site for K238Y and K238F is less prone to interact with oligonucleotide. As a result, both K238Y and K238F accelerate the traversing speed of oligonucleotide through aerolysin. Interestingly, the translocation for (dA_4_) in K238Y is obviously 1.5 times longer than that in K238F (Figure S6), which verify the results that hydrogen bonds is one of the most important factors for the translocation process of oligonucleotides through aerolysin nanopore.

## Conclusions

In conclusion, our work investigated further into the sensing mechanism for the sensing interface of the aerolysin nanopore. The K238C mutant aerolysin which could form stronger hydrogen bonds with oligonucleotides represented as prolonged duration time compared with K238G and WT at +120 mV. Similarly, the amino acid tyrosine of K238Y mutation owing to the hydroxyl leads to a longer dwell time than K238F. These findings determine that the hydrogen bond as one of the important effects attributed to the non-covalent interactions between oligonucleotide and single aerolysin nanopore sensing interface, but not the dominated effect. The complicated analyte-pore interactions including van der Waals, electrostatic interactions, and hydrophobic effect jointly affect translocation behavior. These insights give detailed information for rationally designing the sensing mechanism of the aerolysin nanopore, thereby providing deep understanding for the weak interactions between biomolecules and the confined space for nanopore sensing.

## Data Availability

All datasets generated for this study are included in the manuscript/Supplementary Files.

## Author Contributions

M-YL designed and carried out the nanopore experiments, performed image processing, interpreted data, and wrote the manuscript. Y-QW performed proaerolysin production and nanopore experiments, analyzed data, and wrote the manuscript. YL analyzed data, performed image processing, and wrote the manuscript. Y-LY conceived the idea, interpreted data, and wrote manuscript. Y-TL provided substantial contributions to study design, interpreted data, and wrote manuscript.

### Conflict of Interest Statement

The authors declare that the research was conducted in the absence of any commercial or financial relationships that could be construed as a potential conflict of interest.
