# Supplementary material for: Single Molecule Study of Hydrogen Bond Interactions Between Single Oligonucleotide and Aerolysin Sensing Interface
Source: Front Chem. 2019 Jul 31;7:528. doi: 10.3389/fchem.2019.00528 (PMC6684785; doi:10.3389/fchem.2019.00528)
Supplement: Supplementary file 1 [file Data_Sheet_1.PDF]

# *Supplementary Material - Single Molecule Study of Hydrogen Bond Interactions between Single Oligonucleotide and Aerolysin Sensing Interface*

**Table of Contents**


**Figure S1**

Scatter plots of $(dA)_4$ by K238Y mutant aerolysin at different voltages.

**Figure S2**

Scatter plots of $(dA)_4$ by K238C mutant aerolysin at different voltages.

**Figure S3**

Effects of the applied voltage on the duration of $(dA)_4$ by WT, K238G, K238F, K238C and K238Y aerolysin.

**Figure S4**

Duration time histograms of $(dA)_4$ by K238C mutant aerolysin at different voltages.

**Figure S5**

The voltage-dependent duration for $(dA)_4$ translocating through K238C aerolysin nanopore at pH 7.5.

**Figure S6**

Duration time histograms of $(dA)_4$ by K238Y mutant aerolysin at different voltages.

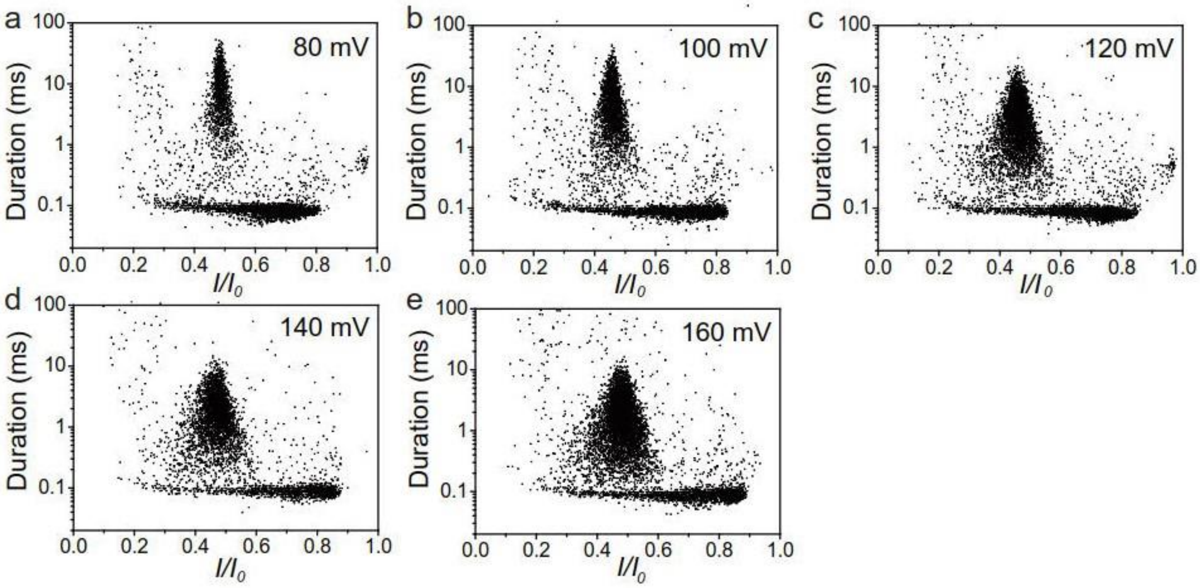


**Supplementary Figure 1.** Scatter plots of $(dA)_4$ by K238Y mutant aerolysin at potential of (a) +80 mV, (b) +100 mV, (c) +120 mV, (d) +140 mV and (e) +160 mV. The data were acquired in 1.0 M KCl, 10 mM Tris, 1.0 mM EDTA, pH 8.0 and in the presence of 2.0 µM $(dA)_4$.

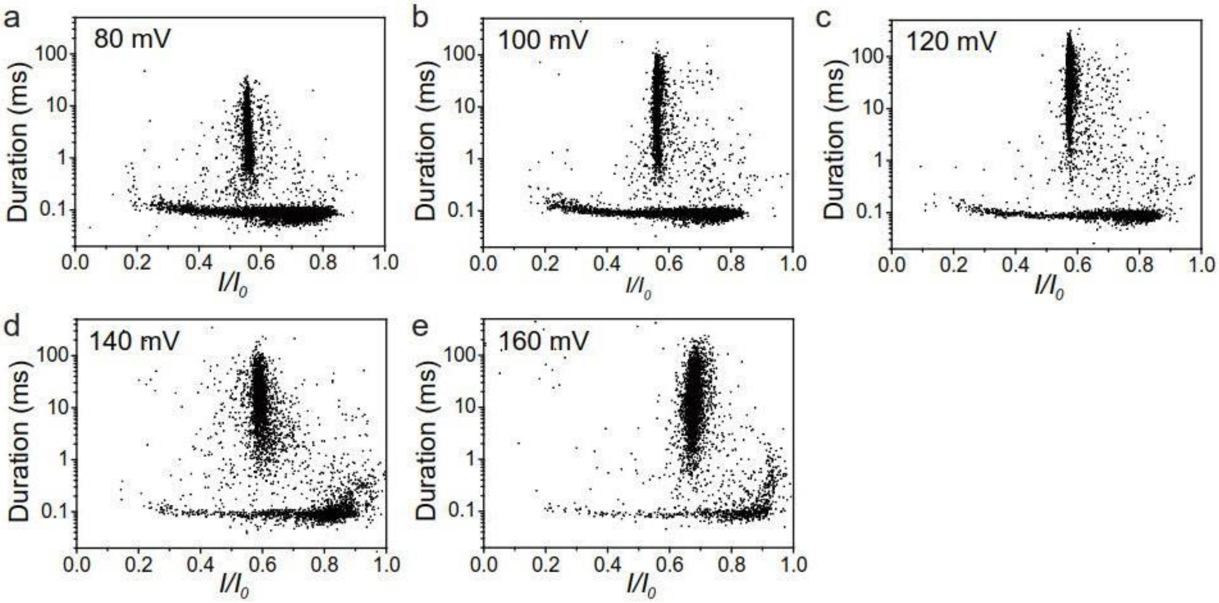


**Supplementary Figure 2.** Scatter plots of $(dA)_4$ by K238C mutant aerolysin at potential of (a) +80 mV, (b) +100 mV, (c) +120 mV, (d) +140 mV and (e) +160 mV. The data were acquired in 1.0 M KCl, 10 mM Tris, 1.0 mM EDTA, pH 8.0 and in the presence of 2.0 µM $(dA)_4$.

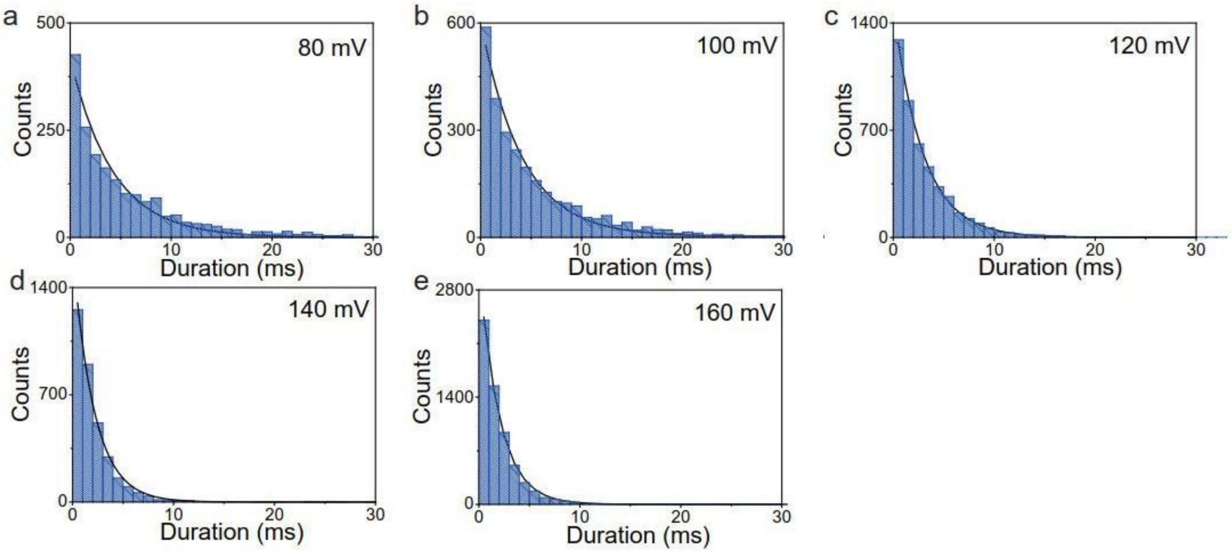


**Supplementary Figure 3.** Duration time histograms of $(dA)_4$ by K238Y mutant aerolysin at potential of (a) +80 mV, (b) +100 mV, (c) +120 mV, (d) +140 mV and (e) +160 mV. All of the histograms were fitted to single Exponential function. The data were acquired in 1.0 M KCl, 10 mM Tris, 1.0 mM EDTA, pH 8.0 and in the presence of 2.0 µM $(dA)_4$.

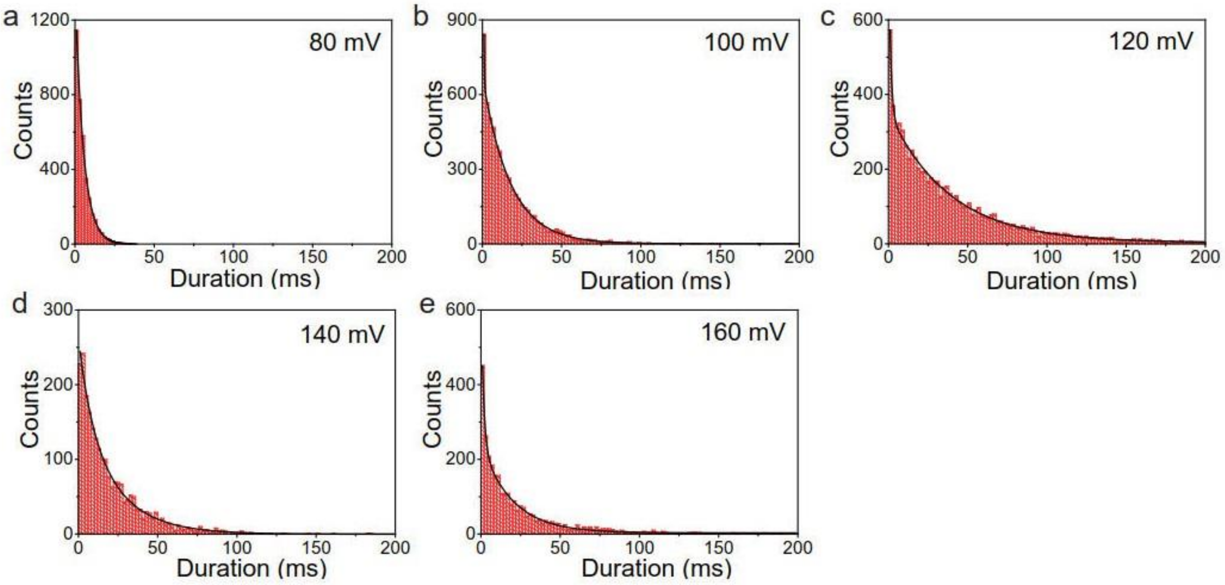


**Supplementary Figure 4.** Duration time histograms of $(dA)_4$ by K238C mutant aerolysin at potential of (a) +80 mV, (b) +100 mV, (c) +120 mV, (d) +140 mV and (e) +160 mV. All of the histograms were fitted to single Exponential function. The data were acquired in 1.0 M KCl, 10 mM Tris, 1.0 mM EDTA, pH 8.0 and in the presence of 2.0 µM $(dA)_4$.

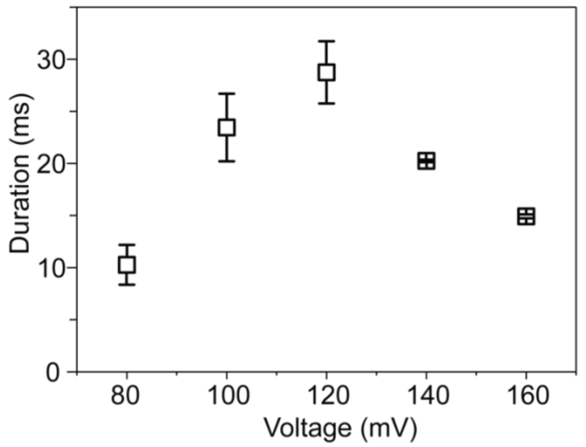


**Supplementary Figure 5.** The voltage-dependent duration for $(dA)_4$ translocating through K238C aerolysin nanopore at pH 7.5. The applied voltage ranging from +80 mV to +160 mV at 20 mV increments. The error-bars indicated standard deviation from data derived from three independent experiments.

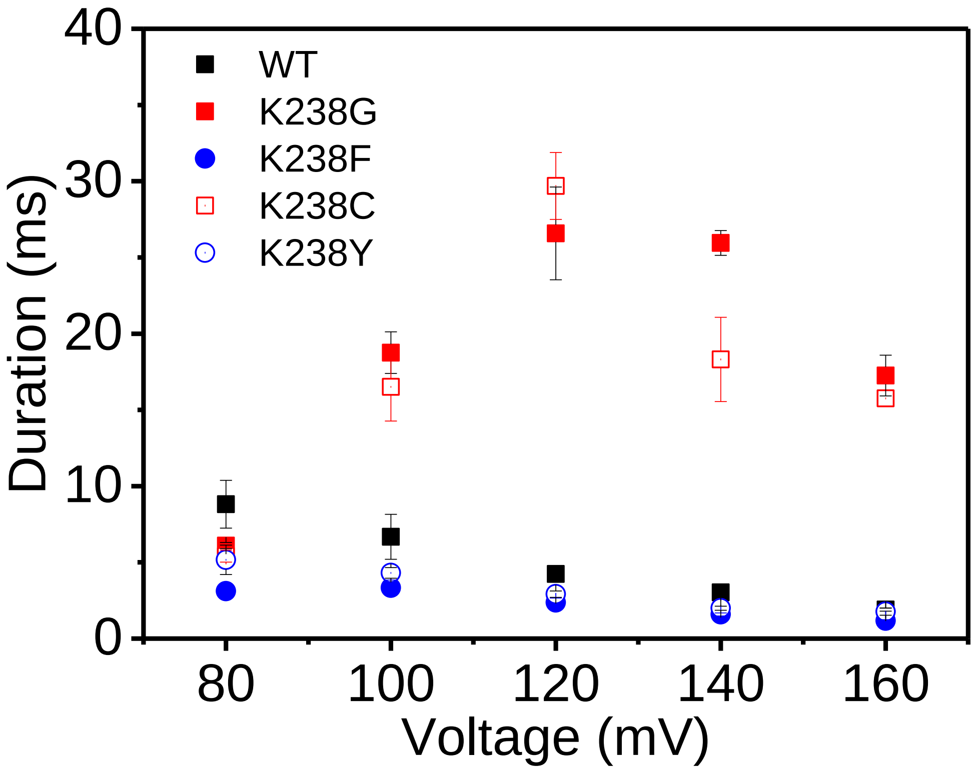


**Supplementary Figure 6.** Effects of the applied voltage on the duration of $(dA)_4$ by WT, K238G, K238F, K238C and K238Y aerolysin, respectively. (excluded bumping events) The applied voltage ranging from +80 mV to +160 mV in -20mV increments. The error-bars indicated standard deviation from data derived from three independent experiments. The data were acquired in 1.0 M KCl, 10 mM Tris, 1.0 mM EDTA, pH 8.0 in the presence of 2.0 μM $(dA)_4$. The data of K238G and K238F is reproduced with permission from Ref. 1. Copyright 2018, American Chemical Society. The data of WT is reproduced with permission from Ref. 2. Copyright 2018, Nature Publishing Group.

**Reference**

[1] Wang, Y.-Q.; Li, M.-Y.; Qiu, H.; Cao, C.; Wang, M.-B.; Wu, X.-Y.; Huang, J.; Ying, Y.-L.; Long, Y.-T. *Anal. Chem.* **2018**, *90*, 7790-7794.

[2] Cao, C.; Ying, Y.-L.; Hu, Z.-L.; Liao, D.-F.; Tian, H.; Long, Y.-T. *Nat. Nanotechnol.* **2016**, *11*, 713 −718.
